# Stakeholder Consensus on Strategies for Collaboration Between Traditional and Biomedical Mental Health Services in Post-Conflict Tigray, Ethiopia

**DOI:** 10.3390/ijerph23020178

**Published:** 2026-01-30

**Authors:** Kenfe Tesfay Berhe, Hailay Abrha Gesesew, Lillian Mwanri, Paul R. Ward

**Affiliations:** 1 College of Health Sciences, Mekelle University, Mekelle, Tigray, Ethiopia; hailushepi@gmail.com; 2 Research Centre for Public Health, Equity and Human Flourishing, Torrens University Australia, Adelaide, SA 5000, Australia; lillian.mwanri@torrens.edu.au (L.M.); paul.ward@torrens.edu.au (P.R.W.); 3 Tigray Health Research Institute (THRI), Mekelle, Tigray, Ethiopia

**Keywords:** traditional healing, biomedical care, collaborative strategies, mental health care, nominal group technique (NGT), post-conflict Tigray, Ethiopia

## Abstract

Ongoing conflicts in sub-Saharan Africa negatively affect the population’s mental health and weaken health care systems. Collaboration among stakeholders is recommended to strengthen mental health services in post-conflict settings, despite limited evidence on context-specific strategies. This paper aimed to identify strategies for collaboration between traditional and biomedical services to improve mental health care. An adapted nominal group technique was employed during a one-day stakeholder workshop. Fourteen participants representing traditional and biomedical mental health services discussed and prioritised strategies based on importance and feasibility to reach consensus. Five collaborative care strategies were prioritised based on stakeholder consensus regarding importance and feasibility: (1) collaborative learning, (2) formalising coordination, (3) capacity building, (4) joint intervention programs, and (5) regulatory support. Key mechanisms for implementing these strategies were also identified, including piloting integrated interventions, appointing a dedicated focal person to coordinate, providing basic psychosocial counselling skills, reducing harmful practices, and strengthening supportive supervision. Mutual learning was identified as a crucial cross-cutting component of all approaches. The conclusion was that implementing these prioritised strategies could improve mental health care. Further research is needed to evaluate the effectiveness of these strategies in enhancing collaborative care and improving mental health outcomes for individuals.

## 1. Introduction

Mental health care services in low- and middle-income countries (LMICs), including in Ethiopia, remain severely limited, with an estimated 75–90% of individuals not receiving adequate treatment [1]. Consequently, in LMICs, such as, Ethiopia, traditional care is used more frequently than biomedical mental health care. More than 70% of people with mental health problems in sub-Saharan Africa (SSA) use traditional healing as their first point of care [2]. In Tigray, before the recent conflict, 68% of individuals with chronic non-communicable diseases were found to use religious holy water as treatment, and nearly half of those who visited healing sites experienced mental health problems [3]. The biomedical services shortage in SSA is mainly due to multiple factors, including the shortage of health care infrastructure, inadequate number of professionals to provide the services [1] and associated sociocultural challenges [4]. The WHO estimates a ratio of 1:500 traditional healers to the population compared to 1:40,000 doctors/population across SSA [5].

Although studies have suggested strategies for collaboration in SSA [6,7,8], available evidence remains very limited, where strategies need to be context-specific [9,10,11]. Additionally, the WHO Traditional Medicine Strategy 2014–2023 also recommends collaborative care of traditional healing as a complement to improved health and patient autonomy [12]. The Ethiopian Health Sector Transformation Plan II (HSTP II, 2020/21–2024/25) recommended implementation activities towards integration of traditional medicine into primary health care [13]. To improve coordinated mental health services, especially in post-conflict settings, it needs guidance on how to engage different stakeholders and mobilise volunteer activities [14]. However, the document lacks clear information on how biomedical health practitioners could collaborate with traditional health practitioners in the Ethiopian setting.

Relying exclusively on traditional care can result in missed or delayed access to biomedical care, and the absence of collaborative care can negatively impact the mental health outcomes of individuals [2,15]. Utilisation of traditional healing services alone causes delays in seeking biomedical services and increases harmful effects [16,17]. Delays in accessing biomedical care can lead to prolonged untreated symptoms, worsened prognosis, and poor medication adherence [17,18,19,20].

Evidence indicates that both traditional healers and biomedical practitioners generally recognise the potential benefits of collaborative mental health care and express willingness to work together [21]. However, translating this willingness into practice remains challenging due to persistent barriers, including differing explanatory models of mental illness, concerns about patient safety, limited scientific evidence supporting traditional practices, and weak referral and communication systems [11,22]. A lack of mutual trust, respect, and knowledge sharing between practitioners has been widely identified as a major obstacle to collaboration, often reinforced by inadequate engagement and communication [11,23]. The absence of robust evidence regarding the effectiveness and safety of traditional healing further contributes to scepticism among biomedical practitioners [24]. Moreover, the health care systems often collapse significantly in conflict areas, leading many communities to rely heavily on community-based interventions, including traditional mental health care [11,25,26].

Therefore, advocating for collaboration between traditional and biomedical practitioners is considered essential to support individuals who require pluralistic health care pathways (6). Collaboration between traditional and biomedical service providers involves working together, where the two parties provide mutual assistance to achieve a common goal (5). Collaborative mechanisms between traditional and biomedical need to balance the context-specific and philosophical healing paradigms of the practices for successful collaboration (7).

The ongoing conflicts in SSA are adversely affecting population health and compromising the functionality of health care systems [27,28,29], leading to increased mental ill health. Consequently, the mental health problems are expected to increase [10,30,31] due to the psychosocial crisis consequences of the war impact [32,33]. However, mental health services are most scarce in conflict settings [34,35,36]. Therefore, a comprehensive mental health care encompassing interventions of medical care, psychological counselling, social support, and vocational rehabilitation is essential for recovery from mental health problems [37,38].

The WHO also recommends primary-health-care-level interventions, including the Mental Health Gap Action Program (mhGAP), and community-based services to strengthen mental health services in conflict settings [39,40]. Community-based psychosocial support mechanisms are required to address the gap in mental health services in conflict-affected settings, including recommendations to work with traditional and religious healers [41]. The WHO [12] and humanitarian guidelines for conflict settings (16) have recognised the importance of traditional healing methods in providing mental health care in low-income countries. Moreover, evidence indicates that traditional healers can provide psychosocial interventions, particularly for common mental disorders such as depression and anxiety, especially with mild symptoms [42,43].

Ethiopia, including the Tigray region, is among the WHO member states that have regulations and registration directives for traditional medicines [44]. However, there are no clear context-specific and stakeholder-consensus-based strategies for collaborative mental health care in the study area. Therefore, the study underpinning this paper aimed to identify the most important and feasible strategies for coordinated care between traditional and biomedical services in the context of mental health. While this study is grounded in the geographical and cultural context of Ethiopia, situated in a post-conflict setting with a therapeutic focus on mental health, the strategies outlined are intended to be transferable to similar contexts beyond those in which the research was conducted.

## 2. Methods

### 2.1. Study Setting and Period

The study was conducted in Mekelle city, Tigray, using an adapted nominal group technique (NGT) that employed face-to-face personal discussions in a workshop. Tigray is one of the 11 federal regions of Ethiopia and comprises seven administrative zones. Before the onset of the war, the Tigray public health system comprised two tertiary hospitals, 14 secondary hospitals, 24 primary hospitals, 224 health centres, and 741 health posts. Among these facilities, only one tertiary hospital in the region provided inpatient psychiatric services [45]. The workshop was conducted in the context of post-conflict Tigray. The Tigray conflict was a two-year war (from November 2020 to November 2022) between the federal government and the Tigray regional government, which resulted in an extensive population and environmental upheaval, leading to significant displacement of communities [46], severe damage to the health system [28] and sexual abuse [47]. Tigray had an estimated population of 7.3 million before the war [48]. The workshop was conducted on 14 November 2024.

### 2.2. Study Design

The NGT is a structured, face-to-face consensus method for prioritising ideas within a feasible session. The method is important in fostering dialogue and understanding across stakeholders with differing cultural frameworks and professional backgrounds [49]. Because it is structured and all partcipants are provided the opportunity to air their idea(s), it helps mitigate the effects of power imbalances and dominant voices, and it is important to consider it when engaging stakeholders with differing levels of social and institutional authority [50]. Therefore, the NGT was considered a reliable approach because it can be completed in a single face-to-face session, does not rely on online platforms, and reduces the risk of participant attrition in the disrupted communication context of post-conflict Tigray. As a qualitative methodology, the NGT was useful in exploring different strategies and their levels of importance and feasibility through prioritasation.

The NGT study design comprised four key steps: silent generation, round robin, clarification, voting using ranking or rating [51], and discussion stages [50]. The NGT is a highly adaptable method, including voting, which may be completed by either allocating a score ranking or by a rating on a Likert scale [50]. The NGT can be adapted to consider people’s educational level [49] and diverse stakeholders [52]. In the process of prioritising, three to five final solutions for a final report was commonly observed in previous studies, although it can vary based on the context of the research and the number of ideas listed [53]. The Consolidated Criteria for Reporting Qualitative Research (COREQ) 32-item checklist was used for the qualitative report [54].

The strategies proritised in this study were informed by preliminary findings from previously published studies within the same research project, which examined factors influencing collaborative care [55], and proposed strategies for collaboration between traditional and biomedical mental health services [56]. These earlier studies were grounded in a collaborative care model for mental health derived from a 2022 systematic review conducted in South Africa [16]. In addition, the social-ecological model was used to conceptualise the multilevel influences on collaborative care [57]. The current NGT study is therefore theoretically informed by a collaborative care framework.

### 2.3. Participants Recruitment

To recruit the participants, a nomination worksheet was developed to categorise stakeholders and identify relevant organisations before nominating the names of the experts/leaders. The worksheet contains the experts’ positions and disciplines, considering the research, policy, clinical practice, experience and managerial positions. A separate worksheet was prepared as per the details of biomedical health care and traditional healing practitioners. Stakeholders were contacted using a letter of invitation one week before the workshop. Given that the workshop was conducted in collaboration with Tigray regional health bureau (TRHB), the invitations were sent through TRHB to the experts. Where there was no response, the selected experts were also communicated with via phone and/or in person.

Panel member recruitment was aimed at a minimum of one participant from each clinical psychiatry practitioner, mental health researcher, traditional healing practitioner, religious leader, mental health care manager, and biomedical health care program directors/policymakers. The maximum recruitment plan for the NGT workshop was to recruit up to 20 participants.

### 2.4. NGT Process

A one-day workshop was conducted on 14 November 2024 in Mekelle, Tigray, with fourteen traditional and biomedical services of mental health service stakeholders. The workshop was facilitated by a senior mental health professional among the research members, a religious leader, and public health experts with senior experience in qualitative research. The discussion was held in Tigrigna, where each audio conversation was recorded. We used adapted NGT ranking stages, and round numbers and ratings were considered in relation to the participants’ educational backgrounds and diversity.

**Figure 1:**
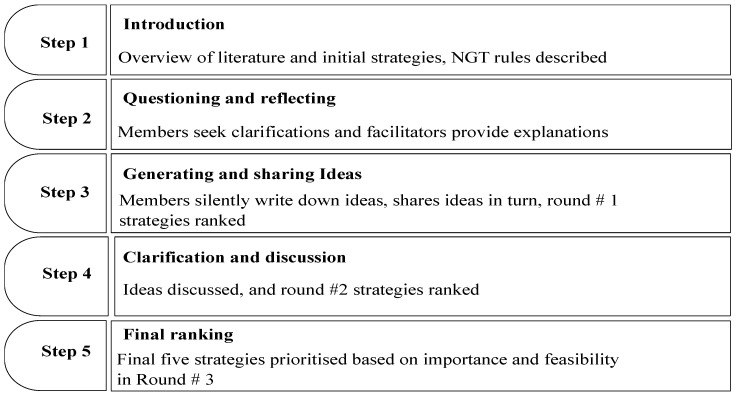
Adapted flowchart of the nominal group technique process.

A summary of the NGT process is described below. The NGT process includes five steps: (a) introduction presentation of suggested strategies from a literature review, (b) questioning and reflecting on the presentation of initial strategies, (c) generating ideas and round 1 ranking based on importance, (d) discussion and round 2 ranking based on importance, (e) final round 3 ranking based on feasibility and consensus on the top-five-ranked prioritised strategies based on importance and feasibility. The adapted NGT process of the current study is presented in Figure 1.

The detailed NGT process is described as follows:

- Introduction: A summary of the suggested strategies was presented, focusing on traditional healing effectiveness [17], the need and influencing factors of collaborative care, and suggested strategies for collaborative care between traditional and biomedical services from preliminary findings of the published papers of a research project [55,56].
- Questioning and reflecting: Participants were asked for clarifications following the presentation on each of the initial strategies listed, and then facilitators made minor editorial corrections according to the feedback from the panel members.
- Generate and share ideas (round 1): Participants were provided time to silently generate ideas independently to state additional strategies. The facilitator then asked one participant at a time to state a single idea to the group in a round-robin fashion and share ideas with the group. Then, the facilitators ensured everyone understood the list of strategies clearly. Ideas were described and recorded verbatim on a flipchart to identify duplicates and newly generated strategies. Accordingly, there was a modification of the strategy statements. For example, mobile clinic was changed to an outreach service; integrated intervention at the holy water site was modified to include both the holy water and the biomedical rehabilitation centres. However, no additional new strategy emerged during this stage.
  Then, participants were asked to provide ratings, and they rated all the initially 13 listed strategies in order of their importance using a 1–5 Likert scale, with 1 representing very little importance and 5 representing the highest importance. The rate value was summed in Excel, ranked in ascending order of importance, and then projected on the screen to the members. Finally, the initial strategies’ ranking score was produced, and the top 10 strategies were shortlisted for the next round rating, round 2.
- Clarification and discussion (round 2): Clarification and discussion were again conducted on the 10 ranked levels of the strategies. Discussions were conducted through facilitators’ probing questions about the benefits of the strategies, how the strategies can be implemented, what factors they think influence the implementation of collaborative mental health care, and how barriers can be addressed to promote collaborative care between traditional and biomedical mental health care in Tigray. Consequently, based on a Likert scale of 1–5 rating, five prioritised strategies were again identified based on their importance in round 2.
- Final ranking (round 3): Participants were then asked to rank the five strategies based on feasibility (using the Likert scale of 1–5) in round 3. The facilitators opened the chance for additional clarifications on the results of the ranked strategies and for any disagreements. Finally, panel members endorsed the five important and feasible strategies separately in ranking order.

### 2.5. Research Positionality and Reflexivity

The primary author (KTB) was one of the facilitators of the NGT, alongside a religious leader and an experienced qualitative researcher. KTB is a mental health clinician in the study area with experience in both clinical psychiatry and community-based mental health services engagement. KTB also occupies a dual position as a biomedical professional and as an individual affected by the same conflict context, which may have introduced potential bias. To address this, KTB maintained a reflexive guide to monitor personal assumptions, emotional responses and interpretive influences throughout the research process. All members of the research team possess strong expertise in qualitative research methods. Regular supervision from senior members of the research team (PW, LM and HAG), together with peer debriefing sessions, was used to minimise bias and enhanced the trustworthiness of the findings [58].

### 2.6. Data Management and Analysis

The initial list of strategies and the Likert scale scoring sheet for ranking were translated into Tigrigna prior to the workshop by a member of the research team who is a mental health expert. The translation was subsequently reviewed by two additional mental health experts fluent in Tigrigna. The recorded discussion points were labelled under each strategies using the NVivo Qualitative Data Analysis Software, version 15.1.1 [59]. The recorded Tigrigna audio was first transcribed and then translated into English. Two research team members (KTB and HAG), fluent native speakers of Tigrigna, carried out the transcription, translation and coding. The other team members (PW and LM) reviewed the documents for accuracy and clarity.

A content analysis approach [60,61], involving the systematic examination and interpretation of the textual content from workshop members who discussed ideas on implementation strategies and their mechanisms, was used to identify and summarise meanings. The ideas were coded under each of the prioritised strategies for collaborative care. The systematic content analysis process involved transcribing and reviewing data, coding and classifying information, identifying strategy-related themes, and revisiting all steps to ensure that the categories were comprehensive and well-defined [61].

Data analysis involved qualitative and quantitative components, with triangulation achieved through the integration of discussion and ranking data, which are presented in tables. The content analysis was used for the qualitative component, including participants’ justifications for rankings across the three rounds and ideas generated during the discussion sessions. The analysis focused primarily on the five prioritised strategies identified. Qualitative data for the analysis were derived from audio-recorded group discussions and field notes taken during the sessions. To ensure anonymity in data management, ranking was conducted individually using separate printed ranking sheets identified using participant codes. Summary ranking results were compiled and presented to participants by the facilitators without identifying individual responses.

### 2.7. Ethical Consideration

Selected expert stakeholders were given an information sheet to understand more about the aim of the study and a consent form to sign to confirm their willingness to participate in the workshop. Ethical clearance was granted by both the Human Research Ethics Committee of Torrens University Australia (0335, 5 September 2024) and the Ethics Review Board of the College of Health Sciences at Mekelle University (MU-IRB 2146/2024, 22 January 2024).

The facilitators informed the participants about the confidentiality and security of the data. Participants were advised not to mention the locations of traditional or biomedical health care services and not to disclose any personal identifiers. In addition, the regulatory office was excluded from attending the workshop.

### 2.8. Operational Definitions

Collaborative care is operationally defined as professionals working closely together to deliver patient care. However, it does not imply ‘integration’, which, as an organisational framework, involves a single entity overseeing and delivering coordinated patient care. Integration requires collaboration as a precondition, but collaboration does not require integration [62].

Mental health problems encompass health conditions characterised by unexpected disturbances in a person’s cognition, emotions, and behavioural control, preventing them from functioning effectively [63]. In biomedical services, core terminologies such as mental health problems, mental illness, mental disorder, and mental/psychological distress were measured using validated psychiatric rating scales, and non-specific psychological or behavioural complaints were considered in our research project. Therefore, we preferred to use the term ‘mental health problems’ in this research, as it is a broader term that encompasses all these concepts.

Traditional healing is defined based on experiences indigenous to different cultures and usually has a long history [12]. Traditional healers’ practices include using holy rituals, divination, and prayer, and sometimes they are combined with a herbal healing modality to treat mental health problems [42].

Biomedical care is a term commonly referred to as modern, Western, conventional, and allopathic in various studies and reviews. There are no clear reasons for the use of those different terms in different studies. This research uses biomedical terminology in the area of traditional and biomedical mental health care reviews in the LMIC setting. Therefore, we prefer to use the nomenclature biomedical health service and biomedical health professionals [21].

## 3. Result

### 3.1. Characteristics of Stakeholders

Fourteen participants, including one policymaker, one Clinical service coordinator, two mental health directors, one psychiatry clinician, one mental health researcher, two religious leaders and six traditional healers, attended the workshop. The details of the panel members’ demographic characteristics are described in Table 1.

### 3.2. Prioritised Strategies of Collaborative Care

After discussion and explanations, participants agreed on the importance of collaborative care. Workshop participants assured that the prioritised strategies were in line with their discussion. The operational definitions of the strategies are presented in Table 1, along with their scores based on a 1–5 Likert scale, where 1 indicates very low importance/feasibility and 5 indicates the highest importance/feasibility as rated by each participant. The operational definitions of importance and feasibility used for rating were as follows: importance refers to the anticipated impact of each strategy on achieving the implementation of collaborative care, while feasibility refers to the practicality of implementing each strategy within the existing system, considering factors such as financial capacity, technical resources, cultural and religious contexts, and relevant policies that may influence collaborative care.

In round 1, 10 of the 13 strategies were prioritised based on their importance. The three highest-ranked strategies were formalising coordination, capacity building, and the deployment of para-counsellors, as shown in Table 2. In round 2, those ten strategies were reranked based on importance, and five were prioritised according to their ranking order (Table 3). In round 2 (Table 3), the top five strategies prioritised for importance were collaborative learning, formalising coordination, capacity building, regulatory support, and joint intervention programs. Table 4 presents the round 3 ranking of the top strategies based on feasibility. The order of prioritisation from highest to lowest feasibility was collaborative learning, formalising coordination, capacity building, joint intervention programs, and regulatory support.

For each round of the nominal group technique, the total score for each strategy was calculated by summing the 1–5 Likert-scale ratings provided by all participants, where 1 indicated very low importance or feasibility and 5 indicated very high importance or feasibility. In rounds 1 and 2, strategies were rated based on importance, while in round 3 they were rated based on feasibility. The detailed narrative summary presents the key discussion points related to the five top-ranked strategies prioritised for both importance and feasibility.

### 3.3. Collaborative Learning

The collaborative learning strategy was ranked first in both importance and feasibility scores. Members strongly suggested that the collaborative learning process should include opportunities to visit healing sites, rather than being limited to formal meetings. There was an intense discussion on setting agendas for mutual learning between the religious and biomedical members of the panel, as described below, especially regarding the standard of holy water treatments. Setting clear agendas for regular meetings was recommended as a foundation strategy of one of the mechanisms to improve collaborative care through sharing experiences and mutual learning. Conducting regular review meetings was mentioned as a way to help government administrators make decisions based on proper consultations with stakeholders. Participants were supported in conducting regular workshop meetings as the main platform for experience sharing to improve knowledge of collaborative care and evaluate the status of its implementation. Participants on the biomedical side admitted that some patients need both biomedical and spiritual treatment, and joint discussions were seen as opportunities to address clients’ needs through collaborative care.

Participants proposed that review meetings should be conducted regularly, engaging practitioners, administrators, patients, families, and community representatives, which was seen as vital for sustaining collaboration and resource mobilisations. The selection and focus of agendas before conducting the review meetings were debated, and they said the agendas should focus on client benefits and the sustainability of collaborative work. One example of an agenda raised by the religious leader to be discussed was the concerns about unregulated individuals operating in holy water sites without proper authorisation and providing inappropriate services that were believed to have damaged the reputation of religious institutions. In another example, the need to introduce Bible-based spiritual counselling standards in holy water sites was raised as an agenda item to frame context-specific spiritual counselling guidance and to communicate with both practitioners as a standard. A workshop member believes that this mechanism can minimise harmful practices and can create long-lasting collaboration as it can create trust, and the recommended idea is stated below:

*“…, I recommend that the holy water supporters be guided by Bible-based or biblical spiritual counselling standards to collaborate with them comfortably. Of course, we can work together for a short-term plan to support the clients, but to improve collaborative care, considering its sustainability, we need to discuss the possibility of introducing the bible based spiritual counselling services in the holy water sites” (P8, Biomedical Clinical service coordinator)*.

One holy water treatment site’s religious treatment coordinator believed that the above concern could be raised, as some traditional healers work in some holy water sites without permission from religious administrative offices. The religious healing coordinator stated the situation:

*“…, even we ourselves from the religious side, we call them they are parasites to our church, they are working without permission and providing inappropriate services, …, there are some individuals who call themselves traditional healers, arrange divorces for people in love for the sake of money by making prolonged follow-ups, and this is damaging the church’s reputation. Therefore, such things can be solved through discussions” (P13, Traditional healing coordinator).*

### 3.4. Formalising Coordination

Formalising coordination was the second-ranked collaborative care strategy in terms of its importance and feasibility among panel members. The members described the importance of having a responsible focal person and a clear guide for coordination. The issue of appointing a mental health expert at the regional health bureau level to lead the mental health services of the mainstream system of the region and incorporating the collaborative care responsibility with the same office was boldly mentioned during the discussion.

The strong recommendation of appointing a mental health expert as a focal person at the regional health bureau level was agreed as a critical mechanism for effective coordination implementation. The members believe that mental health experts have a better understanding of the benefits of comprehensive mental health care by engaging traditional healers. Without this, some members described even the existing biomedical services as being poorly coordinated and documented due to inadequate professional administration. The idea of assigning senior mental health professionals in coordination was described as aiming to have an expert focal person with the responsibility of focusing on improving mental health services, including collaborative care, rather than merging with non-communicable disease coordination in the existing coordination position, which creates overburden and weakens service provision quality. They also argued that mental health services should be led by professionals in the field, just as how a pharmacy is led by pharmacists. A panel member from the biomedical side stated:

*“There is a focal person for mental health integrated with the non-communicable disease (NCD) coordinator [at the regional health bureau level], but it is led by General practitioners (GP), not mental health experts. Given that the NCD coordinator is also the focal person for mental health, so mental health care may be considered as a secondary responsibility for the coordinator, seeking limited attention. But if the focal person assigned at the regional level is a mental health expert, collaborative care may be smoothly coordinated within the mainstream health system” (P6, Policy maker).*

The panel members also recommended utilising the Zonal Orthodox Church Administration Council offices as entry points to decentralise coordination activities, particularly for engagement with the holy water healing sites. The idea of working closely with the Church office, particularly with the training and prayer sections, was believed to facilitate smooth collaboration. In addition, engaging with traditional healers’ associations through their chairmen was also suggested. Assigning focal people at a zonal religious administration office was also stated by one panel member participant as follows:

*“…, I believe it is possible to assign a focal person for the collaborative work at the zonal level of religious offices after starting communication and discussing with the zonal religious administrators.” (P12, Religious Leader).*

The ownership of coordinating resource mobilisation was also mentioned as another responsibility that the members agreed to be integrated and led by the regional health bureau. A panel member noted that since traditional medicine is part of the WHO strategy, the health bureau could play the coordinating role of both services:

*“Regarding coordination and budget mobilisation, as traditional medicine is one component of the WHO strategy, the regional health bureau should coordinate and prioritise activities by engaging all the stakeholders” (P1, Mental health director).*

Although all members of the workshop from both the traditional and biomedical sides agreed to assign the responsibility of coordination to the regional health bureau, and most also agreed to give the mandate for resource mobilisation to the bureau, one member from the traditional healing side disagreed. The member stated the disagreement as follows:

*“…, We [traditional healers] are from different organisational systems. Therefore, the regional health bureau office could organise us, provide recognition and support to both systems’ service quality. However, I believe that budget mobilisation should be mobilised and coordinated separately, as the regional health bureau cannot manage our budget” (P13, Traditional healing coordinator).*

### 3.5. Capacity Building

The lack of capacity in mental health assessment, basic psychosocial support provision skills, patient safety and the lack of instruments, particularly laboratory machines to test the herbs, were among the issues discussed. Suggestions were made for capacity building to be considered as an effective strategy for collaborative care. In addition, a lack of sanitation for patients staying for a few days during treatment was reported, particularly in holy water sites. They emphasised the need for support from the health bureau to improve the quality of patient care, as the religious healing coordinators cannot achieve such improvements alone. A training mechanism was consistently suggested to build the capacity of practitioners to enhance collaborative care. Providing training sessions for traditional healers was seen as an essential step to improve their practice, minimise harmful practices and ensure patients receive safer treatments. Concerns were raised that some practitioners considered the harmful practices in holy water sites to be religious practices. It was therefore suggested that when harmful practices continue after providing training and awareness, mainly such as burning and beating, it should be addressed through legal measures and clear punishments.

*“some religious practitioners in the holy water believe that their harmful practice is religious. But we can minimise dehumanising, harmful practices such as burning and beating of the patients through training, and if the practice continues after awareness provision, it should be governed by legal means and with clear punishments, and I believe, it is not enough to pass them by creating awareness only” (P1, Mental health director).*

The issue of testing herbal medications was also noted as another need for capacity building. The lack of skills, machines/equipment and corresponding resources to install them for testing the effectiveness of herbal treatments in the region were raised. It was proposed that the issue of purchasing equipment and capacity-building budgets be solved through stakeholder involvement. The stakeholders in this instance could be the traditional healers’ associations, which the regional health bureau would organise. Traditional healers’ participation in this study workshop further indicated their association’s willingness to contribute to capacity building by sharing resources. It was noted that they have associations with large memberships, such as one with 250 members in Tigray. One herbal traditional healer member and member of the traditional healers’ association coordination team pointed out the capacity; however, the absence of an organising body has limited their efforts and called for the regional health bureau to take leadership in guiding and coordinating:

*“We, traditional healers, we have the capacity to contribute to sharing resources for training and other purposes, but we did not get a responsible office to organise us. For example, we have a traditional healers association with a membership of around 250 in our area, so the Tigray regional health bureau can lead and guide us in improving the services through training and buying a machine for the herbal effect test. We have no problem allocating budget to improve our service capacity” (P5, Herbal traditional healer).*

### 3.6. Joint Intervention Program

Although the introduction of integrated interventions was ranked fifth in importance and fourth in feasibility during the prioritisation of strategies, stakeholders reached a stronger consensus regarding its importance. The main concern of the members was the feasibility issue related to the budget, especially regarding opening counselling rooms and personnel costs. Collaborative care through integrated intervention programs between traditional and biomedical mental health care services at holy water sites and at biomedical mental health rehabilitation centres was recommended by the NGT workshop members. The importance of integrated service is believed to provide comprehensive care, improve medication adherence, trace severe cases early, initiate timely biomedical treatment, enable early identification of harmful practices, link clients who need further help and improve access to mental health. They suggest that the integration mechanisms include on-site outreach consultation support, space sharing for the provision of integrated services and sharing mental health care skills. To introduce the integration service at holy water sites, it was suggested that a dedicated trained person be assigned at the sites, who would be trained in both biomedical and spiritual counselling. About one mechanism of integrating the services, the following was a quote from one participant:

*“I believe the church administrators (who have the holy water sites in their compound) should decide to assign specific individual spiritual counsellors who can always be available in the holy water sites, and can be supported by training from the nearby hospitals with regular supervision together with church administrators*” *(P12, Religious leader).*

The emphasis of an integrated approach was suggested to be started during the biomedical mental health student training programs for exposure of biomedical students in traditional healing settings as part of the existing community-based mental health education internship. Deploying students to traditional practice sites was seen as an essential entry point for students’ early exposure to collaborative knowledge and practice and creating opportunities for referrals between the two systems. One member of the workshop recalled the benefit from an attachment program during training, where students visited a traditional healing site and reported their current experience of linking clients who need to combine both religious and biomedical treatments; an example was reported by emphasising that integration would benefit clients even more:

*“I had a female client with a history of sexual abuse following the conflict, after observing her feelings of guilt because of the sexual abuse, I recognised the need for spiritual support to the client, so I sent her to a priest for spiritual support and explained to her, “My daughter, you are not the sinner; the sin belongs to the abuser.” Then, she was convinced that she was not a sinner. As a result, she improved after receiving both spiritual and biomedical support. Therefore, counselling would be beneficial on both sides if we deliver it appropriately and in integration” (P4, Clinical psychiatry professional).*

In terms of service provision, basic psychosocial support services were recommended, given that both systems serve similar clients and could benefit from joint spiritual and counselling support efforts. Practical experiences were shared, including instances in which religious elders were already engaged as spiritual supporters in substance rehabilitation centres in Tigray and witnessed clients’ improvement after combined services. In addition to biomedical experts providing services at traditional sites, it was suggested that experienced spiritual healers should contribute to biomedical rehabilitation care centres and primary health care settings, particularly in the context of post-conflict trauma and rehabilitation, where spiritual support was considered necessary. Outreach service provision was recommended, including an outreach program where one or two professionals could provide it and could use public transport to provide counselling support at holy water sites to make it feasible. An assistant professor in mental health who has experience working as a psychiatry clinician stated the importance of integrated service using outreach services at holy water sites as follows:

*“…, One of the important aspects of it is for drug adherence of clients, as we face the fact that some clients stop taking their medications in the traditional healing sites, stopping the medications prescribed at hospitals. The second advantage of working together is to trace people with severe illness and to start biomedical treatments before the illness gets worse. Therefore, having outreach services by mental health professionals to provide services at the traditional healing sites would have a lot of benefits” (P2, Assistant Professor in Mental Health).*

### 3.7. Regulatory Support

The availability of some budget at the regional health bureau was acknowledged for regulating through onsite supervision of traditional healing sites. However, participants ranked the strategy feasibility last, as they believed the per diem transportation costs would be expensive for the highly dispersed traditional healing sites. Regular supportive supervision as regulatory support was described as a central mechanism to build trust and a service map of traditional healing service sites to enable collaboration between traditional and biomedical service providers.

The issue of harmful practices was discussed to be considered as one component of the aim of regulatory support. Participants argued that awareness creation alone is insufficient to eliminate harmful practices such as burning or beating patients. Instead, legal punishment measures should be in place for those who continue such practices despite awareness efforts. Despite the presence of regulatory guidelines, some participants expressed doubts about their practicability and emphasised that regulations are meaningless without proper enforcement. They mentioned ongoing cases of abuse and called for exemplary measures, which they called violators.

*“some religious practitioners in the holy water believe that their harmful practice is religious. But we can minimise dehumanising harmful practices such as burning and beating of the patients through training, and if the practice continues after awareness, it should be governed by legal means and with clear punishments, and it is not enough to pass them by creating awareness only” (P1, Mental health director).*

Registration and licensing of traditional healers were also highlighted by participants on the traditional healing side, describing them as the basis for regulating services, helping distinguish legal from illegal practitioners, and enabling services to be easily accessible through a clear service map. However, traditional healers described the bureaucratic barriers of navigating between multiple institutions to register their traditional medicine clinic. A traditional healer who has experience in providing mental health services in their traditional medicine clinic stated the bureaucratic barriers of the licensing and patency issue process:

*“The patency confirmation for our herbs is taking very long process. For example, as a traditional healer, we request the regional health bureau for the potency of our herb; they send us to three offices at the regional level, with a lot of misunderstanding among the offices about the steps we should follow. After all these hassles, we were referred to the federal level. At the federal level, it has still not been successful. It is creating a problem in the timely registration of our traditional medicine service and herbs patent” (P7, Herbal traditional healer).*

The existence of traditional medicine guidelines at the regional and national levels was described. According to the guidelines, legal practitioners are those registered to work in a specific location, recognised by their communities, residing in the area for at least three years, endorsed by local community justice councils, and practising safely without harming clients’ mental or physical health, and they should be willing to be supervised by the regulatory office. Illegal practices, including the use of unsterilised equipment, burning, or physical assault, lead to penalties such as fines, imprisonment, cancellation of registration, or closure of the practice area. However, although guidelines exist within regional health bureau regulatory offices, their implementation remains weak, requiring stronger stakeholder and community engagement. The traditional medicine content on who are legal practitioners was described by a policy-maker-level participant as follows:

*“…, legal traditional medicine practitioners are the practitioners who are registered, those who can provide service in a specific place, their service recognised by the local community, living at least three years and above in the local area, receiving the letter of support from the local community Justice council (“Mahberawi Biet Frdi”), safe practices that are not impact the mental and physical conditions of the clients, and volunteer to be supervised for quality of the practices are considered as legal practitioners according the guide we have” (P6, Policy maker).*

## 4. Discussion

The current NGT study is the first of its kind, bringing together the stakeholders of traditional healers, religious leaders, mental health professionals, mental health directors, and policy makers to discuss strategies to improve collaborative mental health service delivery in Tigray. This study provided contextual consensus-based prioritised strategies and recommendations for collaborative care policy and practice to improve the mental health services post-conflict. The stakeholders’ willingness to sit together in the current NGT workshop study could also be an earlier indicator of trust building, indicating that the suggested strategies could help implement collaborative care in Tigray. Although not conducted in Tigray and not an NGT study, one study in Addis Ababa, Ethiopia, provides promising insights into collaboration between religious healers and biomedical practitioners, which led to agreement on collaborative service delivery [64].

International guidance has long emphasised the importance of developing context-specific collaborative approaches to support progress towards universal health coverage, including the appropriate integration of traditional healing practices within formal health systems [12]. The strategies identified in this study are aligned with these recommendations by addressing key domains such as policy and regulation, education and training, research, and service delivery [5,12]. In addition, the findings support broader global initiatives aimed at strengthening the integration of mental health into primary care in low-income settings [65]. The effective implementation of collaborative mental health services requires formalised coordination, alongside regular supervision and structured discussions. Therefore, to ensure the sustainability of collaborative care, coordination needs to begin at the regional level and be decentralised to district areas, as suggested in this NGT study. Through better coordination, collaborative care can be strengthened and better aligned with the cultural preferences approach of service users [8,66].

The strategies suggested in this study are consistent with broader efforts to integrate mental health into primary care in low-income countries via mhGAP through assigning a focal person, supportive supervision and joint learning [39]. The current prioritised strategies are similar to collaborative care between traditional and biomedical services for chronic illness broadly as well in SSA [67,68,69]. In addition, it is promising to pilot the implementation of the strategies as Ethiopia recognises the role of traditional medicine in the health care system of the country [70,71] and as the country is one of the WHO Member States for traditional medicine [44]. Moreover, the suggested strategies are accompanied by descriptions of how stakeholders can implement them, which could increase the likelihood of successful implementation.

Introducing standardised faith-based spiritual counselling was an issue raised by biomedical-side participants of the workshop, who perceived that it could improve trust and collaborative care among the practitioners. This was also supported by review evidence in Africa that faith-based mental health care is believed to improve mental health advocacy and prevent some human rights abuses, improve referral systems, and improve the wellbeing of patients [72]. Spiritual counselling services are considered one component of a comprehensive mental health care [73]. The other strong policy-related recommendation of the members was appointing a mental health expert as a focal person at the regional health bureau level, and a policy-maker-level participant also supported the idea. However, mental health service coordination is currently placed under non-communicable diseases (NCDs) in the health system administration hierarchy [74]. The members believed that mental health experts have a better understanding of the benefits of comprehensive mental health care by engaging traditional healers. This implies that coordination should involve a mental health expert focal person who can better recognise the benefits of engaging traditional healers in collaborative care.

It was suggested that a joint intervention program be implemented at holy water sites by providing basic psychological counselling support through trained religious healers and volunteers. Panel members suggested that integrated service provision at holy water sites could be supported through outreach consultations by health professionals trained in mental health from nearby hospitals. The current study suggested providing counselling services onsite and referring patients who require medication. Our study proposed a referral approach for medication management, unlike the project conducted at a holy water site in Addis Ababa, Ethiopia, where psychiatry clinicians prescribed medications in an onsite clinic within the vicinity of the holy water [64]. This was suggested considering the feasibility issues related to the lack of psychiatry clinicians, for its sustainability, and to address many beneficiaries, as high rates of mental health problems are prevalent in conflict settings [34]. However, as the supportive supervision, licensing, registration, and service mapping of traditional healing service areas are currently fragmented activities [70], it is important to strengthen regulatory support, as recommended in our study.

While this study has strengths, including bringing the core stakeholders to deliberate strategies to improve mental health delivery in Tigray, it has some limitations. In line with typical NGT practice, a small number of experts were recruited purposively, which may have limited the diversity of perspectives represented in the findings. This was due to the study’s focus on expert consensus and prioritisation, the post-conflict setting, and practical constraints related to time and resources, including transport costs, as stakeholders were geographically dispersed and difficult to contact. This approach may therefore have limited the range of perspectives represented. The NGT was conducted in a single session, which was considered appropriate for the study objective and context.

People with lived experience of mental health problems and families/caregivers of patients and the regulatory office were not included as stakeholders in the workshop. The exclusion of caregivers and people with lived experience was another limitation of this study. However, although people with lived experience of mental health problems were not directly involved in the NGT workshop, they participated in preceding studies whose preliminary findings informed the current NGT phase. In particular, people with lived experience who had sought care from both traditional and biomedical services took part in a previously published study [65]. Therefore, the proposed list of collaborative care strategies presented to the stakeholders at the NGT workshop was partly derived from these participants’ perspectives. Regulatory office delegates were excluded because the research team believed it would have been difficult for the traditional healers to speak freely in front of the regulatory body. We obtained documents and regulations from the office to enhance our understanding and discuss the results, but we could not engage them in the NGT workshop. Stakeholders from two zonal administrative areas of the Tigray region did not participate because of the border conflict. Greater discussion depth would have been beneficial if more time had been available; however, as the workshop was limited to one day, opportunities for in-depth discussion may have been constrained.

## 5. Conclusions

The stakeholder consensus on the proposed strategies implies the need to strengthen collaborative care between traditional and biomedical services. The stakeholders underscored the importance of developing culturally responsive and contextually grounded strategies that can address the collaborative care gap. The NGT findings provide prioritised solutions to improve collaboration in resource-limited settings and to contribute to recovery in post-conflict settings. Moreover, our findings support these existing directions and policies, including the WHO Global Traditional Medicine Strategy 2014–2023 and the WHO Comprehensive Mental Health Action Plan 2013–2030, which recommend the integration of traditional healing into community-based interventions and primary health care. Therefore, the findings of this study indicate that mental health systems that operate exclusively within biomedical frameworks are unlikely to meet the pluralistic needs of the people in low-resource and conflict settings. In a post-conflict setting, the implementation of most activities should be integrated within existing post-conflict humanitarian coordination mechanisms involving international and local non-governmental organisations.

The implementation of these prioritised strategies should begin with low-cost and high-feasibility actions, such as structured collaborative learning platforms, referral pathways and clearly defined coordination mechanisms. These approaches can help build trust and shared understanding between practitioners. Concurrently, implementation of capacity-building activities, including joint training, resource sharing, and supportive supervision, can support the gradual development of collaborative intervention programs tailored to local mental health care needs. At the system level, the findings suggest that engagement with policymakers, including the Tigray Regional Health Bureau, and other key stakeholders may support the implementation of collaborative care models. The study highlights the potential value of mental health coordination at the regional level and regular review meetings involving mental health leaders, representatives of traditional healers, religious authorities associated with holy water sites, and biomedical and traditional healing professional associations. Such engagement may help foster supportive regulatory environments and context-specific approaches to collaboration.

Implementing these prioritised strategies may support recovery from post-conflict trauma. Our findings were aligned with Ethiopian and global policy directions on collaborative care for people with chronic illnesses. We recommend further research on the effectiveness of strategies to enhance collaboration between traditional and biomedical services, as well as the mental health outcomes of people in sub-Saharan Africa. While this study is grounded in the geographical and cultural context of Ethiopia, situated in a post-conflict setting with a therapeutic focus on mental health, the strategies outlined are intended to be transferable to similar contexts beyond those in which the research was conducted.

## Figures and Tables

**Table 1:** Demographic characteristics of panel member stakeholders for collaborative mental health care in post-conflict Tigray, *n* = 14.

Code	Sex	Age	Profession
P1	M	40	Mental health director
P2	M	42	Assistant professor in mental health
P3	M	30	Mental health director
P4	M	57	Clinical psychiatry professional
P5	M	39	Traditional healer (mainly herbal)
P6	M	55	Policy maker
P7	M	36	Traditional healer (mainly herbal)
P8	M	36	Clinical service coordinator
P9	M	33	Religious healer
P10	F	41	Religious healer
P11	F	49	Traditional healer
P12	M	39	Religious leader
P13	M	47	Religious healing coordinator
P14	M	49	Traditional healer

**Table 2:** Round 1 rating scores and operational definitions of the thirteen strategies based on importance ranked by stakeholders for collaborative mental health care in post-conflict Tigray, *n* = 14.

List of Initial Strategies	Definition of the Strategies	Sum Score of the 1–5 Likert Scale Rating Based on Importance
Formalising coordination	Formalising coordination of collaborative care by assigning a mental health focal person at the regional health bureau level and in collaboration with stakeholders	60
Capacity building	Capacity building for practitioners and service provision on patient collaborative mental health support and safety	56
Para counsellors deployment	Para-counsellors or basic counselling providers deployment by providing training on screening, providing basic psychosocial support and minimising harmful activities at holy water sites	56
Awareness creation	Awareness creation about the advantages of collaborative care and its mechanisms to the community	55
Collaborative learning	Collaborative learning through regular review meetings and experience sharing among stakeholders on the collaborative care, including practitioners, engaging clients, families and community representatives	54
Regulatory support	Regulatory support to traditional healing services by regional health bureau experts using onsite and/or remote support using technologies	54
Collaborative care guideline	Developing guidelines on collaborative care, incorporating referral pathways	54
Herbal safety and efficacy	Safety and efficacy enhancement mechanisms to prove the effectiveness of herbal medicine healing practices and minimise adverse effects through research	49
Joint intervention program	Integrated intervention program service provision approach through opening counselling and spiritual services in the traditional healing and biomedical rehabilitation centre sites	48
Outreach services	Introducing outreach services in traditional healing and biomedical service sites	45
Licensing traditional medicine	Regular registration and licensing of traditional healers’ practices	44
Resource mobilisation	Resource mobilisation and improving budget allocation for training, awareness, supervision and counselling room renovations	43
Formal education	Formal education by incorporating traditional healing student practice as collaborative care in their curriculum	41

**Table 3:** Round 2 rating scores of the five prioritised (out of ten) strategies based on importance identified by stakeholders for collaborative mental health care in post-conflict Tigray, *n* = 14.

Strategies	Sum	After the Discussion, Rating Scores Using a 1–5 Likert Scale, with 1 Representing Very Little Importance and 5 Representing the Highest Importance by Each Participant													
		P1	P2	P3	P4	P5	P6	P7	P8	P9	P10	P11	P12	P13	P14
Collaborative learning	63	5	4	4	3	5	5	4	4	4	5	5	5	5	5
Formalising coordination	62	5	5	5	4	5	5	4	3	5	5	5	1	5	5
Capacity building	61	4	5	5	2	5	4	5	3	5	5	5	5	3	5
Regulatory support	61	4	5	5	1	5	5	5	5	4	4	5	3	5	5
Joint intervention program	61	5	4	5	2	3	5	4	5	5	5	5	5	5	3
Para counsellors deployment	60	4	5	4	5	3	5	5	5	5	5	4	1	5	4
Herbal safety and efficacy	58	3	5	5	3	4	5	3	5	3	5	5	4	3	5
Outreach services	58	4	5	4	4	1	3	5	5	5	4	4	5	5	4
Collaborative care guideline	58	5	5	5	2	4	4	4	5	3	5	5	2	5	4
Awareness creation	54	3	5	4	2	2	5	5	5	3	5	5	3	4	3

**Table 4:** Round 3 rating scores of the five prioritised strategies based on feasibility identified by stakeholders for collaborative mental health care in post-conflict Tigray, *n* = 14.

Strategies	Sum	After the Discussion, Rating Scores Using a 1–5 Likert Scale, with 1 Representing Very Little Feasibility and 5 Representing the Highest Feasibility by Each Participant													
		P1	P2	P3	P4	P5	P6	P7	P8	P9	P10	P11	P12	P13	P14
Collaborative learning	64	4	5	5	4	5	4	5	5	4	5	5	5	5	3
Formalising coordination	63	5	5	4	5	5	4	5	3	5	4	4	5	5	4
Capacity building	59	5	4	5	5	5	4	4	4	4	3	3	5	5	3
Joint intervention program	56	2	5	3	5	4	3	5	3	4	5	5	5	5	2
Regulatory support	53	4	4	4	4	4	5	4	4	3	3	3	3	5	3

## Data Availability

The original data are available upon request from the primary or corresponding author.

## References

[1] Atewologun F., Adigun O.A., Okesanya O.J., Hassan H.K., Olabode O.N., Micheal A.S., Ahmed M.M., Ukoaka B.M., Idris N.B., Oso T.A. (2025). A comprehensive review of mental health services across selected countries in sub-Saharan Africa: Assessing progress, challenges, and future direction. Discov. Ment. Health.

[2] Williams S.A., Baldeh M., Bah A.J., Dennis F., Robinson D.R., Adeniyi Y.C. (2025). Pathways to mental health services across local health systems in sub-Saharan Africa: Findings from a systematic review. PLoS ONE.

[3] Gebremedhin K.D., Abrha M.W., Tesfamariam T., Gebretnsae H., Wubayoh T. (2024). Characteristic of Holy Water (Mai Tselot) Users in Tigray Region, Northern Ethiopia, Cross-Sectional Study, 2019. J. Arts Humanit. Soc. Sci..

[4] Aguwa C., Carrasco T., Odongo N., Riblet N. (2022). Barriers to Treatment as a Hindrance to Health and Wellbeing of Individuals with Mental Illnesses in Africa: A Systematic Review. Int. J. Ment. Health Addict..

[5] WHO Comprehensive Mental Health Action Plan 2013–2030. https://iris.who.int/handle/10665/345301.

[6] Mbwayo A.W., Ndetei D.M., Mutiso V., Li K. (2013). Traditional healers and provision of mental health services in cosmopolitan informal settlements in Nairobi, Kenya. Afr. J. Psychiatry.

[7] van Rooyen R.M.D., Pretorius B., Tembani N.M., ten Ham-Baloyi W. (2017). Evidence-based recommendations to facilitate professional collaboration between allopathic and traditional health practitioners. Health SA Gesondheid.

[8] Baheretibeb Y., Wondimagegn D., Law S. (2024). “Trust in God, but tie your donkey”: Holy water priest healers’ views on collaboration with biomedical mental health services in Addis Ababa, Ethiopia. Transcult. Psychiatry.

[9] Hamid A.A., Musa S.A. (2010). Mental health problems among internally displaced persons in Darfur. Int. J. Psychol..

[10] Favara M., Hittmeyer A., Porter C., Singhal S., Woldehanna T. (2022). Young people, mental health, and civil conflict: Preliminary findings from Ethiopia’s Tigray region. Psychiatry Res. Commun..

[11] Musisi S., Okello E.S., Abbo C. (2010). Culture and traditional healing in conflict/post-conflict societies. Afr. J. Trauma. Stress.

[12] WHO WHO traditional medicine strategy 2014–2023. https://iris.who.int/handle/10665/92455.

[13] Ethiopian Ministry of Health (2021). Health Sector Transformation Plan II (HSTP II) 2020/21-2024/25. https://extranet.who.int/countryplanningcycles/sites/default/files/public_file_rep/ETH_Ethiopia_Health-Sector-Transformation-Plan-II_2021-2026.pdf.

[14] Tedla M.G., Kahsay M.M. (2023). Mental health rehabilitation and support for victims of the Tigray war. Lancet Psychiatry.

[15] Berhanu K., Birlie A., Fiseha T., Reta Y., Gebreegziabhere Y. (2025). Pathways to psychiatric care in Debre Berhan, Ethiopia: A cross-sectional study. PLoS ONE.

[16] Truter Z.M. (2023). Collaborative care for mental health in South Africa: A qualitative systematic review. S. Afr. J. Psychol..

[17] Berhe K.T., Gesesew H.A., Ward P.R. (2024). Traditional healing practices, factors influencing to access the practices and its complementary effect on mental health in sub-Saharan Africa: A systematic review. BMJ Open.

[18] Girma E., Tesfaye M. (2011). Patterns of treatment seeking behavior for mental illnesses in Southwest Ethiopia: A hospital based study. BMC Psychiatry.

[19] Anjorin O., Hassan Wada Y. (2022). Impact of traditional healers in the provision of mental health services in Nigeria. Ann. Med. Surg..

[20] Wollie A.M., Usher K., Rice K., Islam M.S. (2025). Health Professionals’ Attitudes Towards Traditional Healing for Mental Illness: A Systematic Review. Int. J. Ment. Health Nurs..

[21] Green B., Colucci E. (2020). Traditional healers’ and biomedical practitioners’ perceptions of collaborative mental healthcare in low-and middle-income countries: A systematic review. Transcult. Psychiatry.

[22] Gureje O., Nortje G., Makanjuola V., Oladeji B., Seedat S., Jenkins R. (2015). The role of global traditional and complementary systems of medicine in treating mental health problems. Lancet Psychiatry.

[23] Oseni Z., Shannon G. (2020). The relationship between Indigenous and allopathic health practitioners in Africa and its implications for collaboration: A qualitative synthesis. Glob. Health Action..

[24] Kokota D., Stewart R.C., Abbo C., Bandawe C. (2022). Views and experiences of traditional and Western medicine practitioners on potential collaboration in the care of people living with mental illness in Malawi. Malawi Med. J..

[25] Schulz P., Apio E.O., Oryem R. (2024). Love and Care in the Lord’s Resistance Army (LRA) in Northern Uganda. Glob. Stud. Q..

[26] Musisi S., Sudarmono P., Mollica R.F., Ranieri Guerra M.A.R., Bhasin R., Lavelle J. (2004). Traditional Healing in Conflict/Post-Conflict Societies. Book of Best Practices: Trauma and the Role of Mental Health in Post-Conflict Recovery.

[27] Oladayo N.A. (2019). The impact of conflict on health outcomes: A systematic evidence from sub-saharan Africa. Mgbakoigba J. Afr. Stud..

[28] Gesesew H., Kebede H., Berhe K., Fauk N., Ward P. (2023). Perilous medicine in Tigray: A systematic review. Confl. Health.

[29] Musisi S., Kinyanda E. (2020). Long-term impact of war, civil war, and persecution in civilian populations—Conflict and post-traumatic stress in African communities. Front. Psychiatry.

[30] Gebreyesus A., Gebremariam A.G., Kidanu K.G., Gidey S., Haftu H., Nigusse A.T., Shishay F., Mamo L. (2024). Post-traumatic stress disorder symptoms among internally displaced persons: Unveiling the impact of the war of Tigray. Discov. Ment. Health.

[31] Gebreyesus A., Niguse A.T., Shishay F., Mamo L., Gebremedhin T., Tsegay K., Gebremariam A.G., Kidanu K.G., Gidey S., Tesfay F. (2024). Prevalence of depression and associated factors among community hosted internally displaced people of Tigray; during war and siege. BMC Psychiatry.

[32] Dadi A.F. (2022). The mental health consequences of war in northern Ethiopia: Why we should be concerned. Lancet Psychiatry.

[33] Abreha G.F., Adhanu H.H., Aregawi A.B., Wuneh A.D., Tesfay F., Lema G.K., Demstu B., Teka H., Yemane A., Gidey H. (2025). Exploring physical, sexual and mental health consequences of gender-based violence among women and girls during conflict in Tigray, Ethiopia. BMC Public. Health.

[34] Charlson F., van Ommeren M., Flaxman A., Cornett J., Whiteford H., Saxena S. (2019). New WHO prevalence estimates of mental disorders in conflict settings: A systematic review and meta-analysis. Lancet.

[35] Koebach A., Robjant K. (2021). NETfacts: A community intervention integrating trauma treatment at the individual and collective level. Eur. J. Psychotraumatol.

[36] North C.S., Pfefferbaum B. (2013). Mental health response to community disasters: A systematic review. JAMA.

[37] Colizzi M., Lasalvia A., Ruggeri M. (2020). Prevention and early intervention in youth mental health: Is it time for a multidisciplinary and trans-diagnostic model for care?. Int. J. Ment. Health Syst..

[38] WHO (2019). Recovery Practices for Mental Health and Well-Being: WHO QualityRights Specialized Training: Course Guide. Recovery Practices for Mental Health and Well-Being: WHO QualityRights Specialized Training: Course Guide.

[39] Keynejad R., Spagnolo J., Thornicroft G. (2021). WHO mental health gap action programme (mhGAP) intervention guide: Updated systematic review on evidence and impact. BMJ Ment. Health.

[40] WHO (2021). Facilitating Training of Health Workers on Mental Health Service Provision in Humanitarian Settings. WHO Regional Office for Africa. https://www.afro.who.int/news/who-facilitates-training-health-workers-mental-health-service-provision-humanitarian-setting.

[41] Greene M.C., Getz M., Streicker E., Thind P., Tayama E., Lafto K.M., Ayele N.W., Worku T.A., Carson R., d’Andon C.F. (2025). Strategies to Improve Access to Mental Health and Psychosocial Support Among Displaced Populations in Ethiopia. Curr. Psychiatry Res. Rev..

[42] Nortje G., Oladeji B., Gureje O., Seedat S. (2016). Effectiveness of traditional healers in treating mental disorders: A systematic review. Lancet Psychiatry.

[43] Nwagbo C.A., Moses T. (2022). The effectiveness of traditional healing in the treatment of mental illness in Africa: A critical review. Int. J. Psychother. Afr..

[44] WHO (2019). WHO Global Report on Traditional and Complementary Medicine 2019.

[45] Gebrehiwet T.G., Abebe H.T., Woldemichael A., Gebresilassie K., Tsadik M., Asgedom A.A., Fisseha G., Berhane K., Gebreyesus A., Alemayoh Y. (2023). War and Health Care Services Utilization for Chronic Diseases in Rural and Semiurban Areas of Tigray, Ethiopia. JAMA Netw. Open.

[46] Devi S. (2021). Tigray atrocities compounded by lack of health care. Lancet.

[47] Fisseha G., Gebrehiwot T.G., Gebremichael M.W., Wahdey S., Meles G.G., Gezae K.E., Legesse A.Y., Asgedom A.A., Tsadik M., Woldemichael A. (2023). War-related sexual and gender-based violence in Tigray, Northern Ethiopia: A community-based study. BMJ Glob. Health.

[48] Gesesew H., Berhane K., Siraj E.S., Siraj D., Gebregziabher M., Gebre Y.G., Gebreslassie S.A., Amdeslassie F., Tesema A.G., Siraj A. (2021). The impact of war on the health system of the Tigray region in Ethiopia: An assessment. BMJ Glob. Health.

[49] McMillan S.S., Kelly F., Sav A., Kendall E., King M.A., Whitty J.A., Wheeler A.J. (2014). Using the nominal group technique: How to analyse across multiple groups. Health Serv. Outcomes Res. Methodol..

[50] McMillan S.S., King M., Tully M.P. (2016). How to use the nominal group and Delphi techniques. Int. J. Clin. Pharm..

[51] Delbecq A.L., Van de Ven A.H., Gustafson D.H. (1975). Group Techniques for Program Planning: A Guide to Nominal Group and Delphi Processes.

[52] Cetin-Sahin D., Arsenault-Lapierre G., Bolster-Foucault C., Champoux-Pellegrin J., Rojas-Rozo L., Quesnel-Vallée A., Vedel I. (2024). Building Timely Consensus Among Diverse Stakeholders: An Adapted Nominal Group Technique. Ann. Fam. Med..

[53] Thier M., Mason D.P. (2019). Breaking ranks? Differentiating nominal group technique scoring approaches for consensus and prioritization. Int. J. Res. Method. Educ..

[54] Tong A., Sainsbury P., Craig J. (2007). Consolidated criteria for reporting qualitative research (COREQ): A 32-item checklist for interviews and focus groups. Int. J. Qual. Health Care.

[55] Berhe K.T., Gesesew H.A., Mwanri L., Ward P. (2025). Barriers and Facilitators to Collaboration Between Traditional and Biomedical Mental Health Services in a Post-Conflict Healthcare System: A Qualitative Study in Tigray, Ethiopia. SSM Health Syst..

[56] Berhe K.T., Ward P., Mwanri L., Gesesew H.A. (2026). Strategies for collaborative mental health care in post-conflict Tigray: A qualitative study. SSM Ment. Health.

[57] Sallis J.F., Owen N., Fisher E., Glanz K., Rimer B.K., Viswanath K. (2015). Ecological models of health behavior. Health Behavior and Health Education: Theory, Research, and Practice.

[58] Tisdell E.J., Merriam S.B., Stuckey-Peyrot H.L. (2025). Qualitative Research: A Guide to Design and Implementation.

[59] Lumivero (2025). NVivo Qualitative Data Analysis Software.

[60] Kuckartz U., Radiker S. (2023). Qualitative Content Analysis: Methods, Practice and Software.

[61] Bengtsson M. (2016). How to plan and perform a qualitative study using content analysis. NursingPlus Open.

[62] Boon H.S., Mior S.A., Barnsley J., Ashbury F.D., Haig R. (2009). The difference between integration and collaboration in patient care: Results from key informant interviews working in multiprofessional health care teams. J. Manip. Physiol. Ther..

[63] American Psychiatric Association (2013). Diagnostic and Statistical Manual of Mental Disorders.

[64] Baheretibeb Y., Wondimagegn D., Law S. (2021). Holy water and biomedicine: A descriptive study of active collaboration between religious traditional healers and biomedical psychiatry in Ethiopia. BJPsych Open.

[65] WHO Mental Health Gap Action Programme (mhGAP). https://www.who.int/teams/mental-health-and-substance-use/treatment-care/mental-health-gap-action-programme.

[66] Krah E., de Kruijf J., Ragno L. (2018). Integrating traditional healers into the health care system: Challenges and opportunities in rural northern Ghana. J. Community Health.

[67] Matungwa D.J., Hong R., Kidola J., Pungu D., Ponticiello M., Peck R., Sundararajan R. (2022). Understanding the role of traditional healers in the HIV care cascade: Findings from a qualitative study among stakeholders in Mwanza, Tanzania. PLoS Glob. Public Health.

[68] Ilozumba O., Koirala S., Meka A., Ossai E., Choudhury S.M., Wagner R., Lilford R. (2023). Exploring the possibility of collaboration for biomedical professionals and traditional healers: A Systematic Review. medRxiv.

[69] Jama N.A., Nyembezi A., Ngcobo S., Lehmann U. (2024). Collaboration between traditional health practitioners and biomedical health practitioners: Scoping review. Afr. J. Prim. Health Care Fam. Med..

[70] Demeke H., Hasen G., Sosengo T., Siraj J., Tatiparthi R., Suleman S. (2022). Evaluation of policy governing herbal medicines regulation and its implementation in Ethiopia. J. Multidiscip. Healthc..

[71] Dubale S., Edris R., Abebe E., Kebebe D., Abdissa N., Debela A., Zeynudin A., Suleman S. (2023). Traditional medicine regulatory framework and legal basis in Ethiopia: A critical evaluation of challenges and opportunities for policy implementation. Res. Sq..

[72] Nanji N., Olivier J. (2024). Providing mental healthcare through faith-based entities in Africa: A systematic review. Christ. J. Glob. Health.

[73] Wollie A.M., Usher K., Rice K., Islam M.S. (2025). Challenges and opportunities for integrating traditional healing approaches with biomedical care for mental illness: A scoping review from healers’ perspectives. PLoS ONE.

[74] Hanlon C., Eshetu T., Alemayehu D., Fekadu A., Semrau M., Thornicroft G., Kigozi F., Marais D.L., Petersen I., Alem A. (2017). Health system governance to support scale up of mental health care in Ethiopia: A qualitative study. Int. J. Ment. Health Syst..

